# High resolution optical mapping of cardiac electrophysiology in pre-clinical models

**DOI:** 10.1038/s41597-022-01253-1

**Published:** 2022-03-31

**Authors:** Christopher O’Shea, James Winter, S. Nashitha Kabir, Molly O’Reilly, Simon P Wells, Olivia Baines, Laura C. Sommerfeld, Joao Correia, Ming Lei, Paulus Kirchhof, Andrew P. Holmes, Larissa Fabritz, Kashif Rajpoot, Davor Pavlovic

**Affiliations:** 1 Institute of Cardiovascular Sciences, University of Birmingham, Birmingham, UK; 2 Heart Center, Department of Clinical and Experimental Cardiology, Amsterdam UMC, location AMC, Amsterdam, The Netherlands; 3 William Harvey Research Institute, Queen Mary University of London, London, UK; 4 Department of Pharmacology, University of Oxford, Oxford, UK; 5 Department of Cardiology, University Heart and Vascular Centre, University Medical Center Hamburg-Eppendorf, Germany and German Center for Cardiovascular Research (DZHK) partner site Hamburg/Kiel/Lubeck, Lubeck, Germany; 6 Institute of Clinical Sciences, University of Birmingham, Birmingham, UK; 7 University Center of Cardiovascular Science, UKE, Hamburg, Germany; 8 School of Computer Science, University of Birmingham, Birmingham, UK

**Keywords:** Arrhythmias, Heart failure

## Abstract

Optical mapping of animal models is a widely used technique in pre-clinical cardiac research. It has several advantages over other methods, including higher spatial resolution, contactless recording and direct visualisation of action potentials and calcium transients. Optical mapping enables simultaneous study of action potential and calcium transient morphology, conduction dynamics, regional heterogeneity, restitution and arrhythmogenesis. In this dataset, we have optically mapped Langendorff perfused isolated whole hearts (mouse and guinea pig) and superfused isolated atria (mouse). Raw datasets (consisting of over 400 files) can be combined with open-source software for processing and analysis. We have generated a comprehensive post-processed dataset characterising the baseline cardiac electrophysiology in these widely used pre-clinical models. This dataset also provides reference information detailing the effect of heart rate, clinically used anti-arrhythmic drugs, ischaemia-reperfusion and sympathetic nervous stimulation on cardiac electrophysiology. The effects of these interventions can be studied in a global or regional manner, enabling new insights into the prevention and initiation of arrhythmia.

## Abstract (summary)

**Table:** 

Measurement(s)	Cardiac Action potential • Caridiac Calcium transient
Technology Type(s)	Voltage dye cardiac optical mapping • Calcium dye cardiac optical mapping
Factor Type(s)	Pacing Cycle Length • Ischemia-Reperfusion • Sympathetic Nervous Stimulation • Flecainide • Carbenoxolone • Ibutilide • Cyclopiazonic acid • HMR 1556 • Noradrenaline
Sample Characteristic - Organism	Mouse • Guinea pig

## Background & Summary

Organised depolarisation, propagation, and repolarisation of the cardiac action potential is key to coordinated contraction and relaxation of the heart. Local, regional, or global abnormalities in action potential generation, propagation and repolarisation can disturb the normal rhythm or beating rate of the heart, known as cardiac arrhythmia. Arrhythmias are common and contribute to several cardiovascular complications including sudden cardiac death, heart failure and stroke^1^. The mechanisms underpinning complex arrhythmias, and how to best treat these conditions, remain incompletely understood^2^. This has fuelled a substantial and ongoing global research effort.

Cardiac optical mapping is a fluorescence-based technique which offers unparalleled spatial resolution to study the dynamics of cardiac electrophysiology^3,4^. Using potentiometric dyes, action potential propagation and morphology in multi-cellular cardiac preparations, including *ex-vivo* animal hearts, are visualised. Optical mapping has several advantages over traditional electrode measurements^5^. High spatial resolution enables local and/or regional alterations in cardiac electrophysiology to be observed (e.g. different chambers of the heart, apico-basal gradients)^6,7^. Furthermore, optical mapping enables direct contactless recording of optical action potentials, whereas multi electrode array techniques make indirect recordings of extracellular field potentials which require direct or close electrode-tissue contact^8,9^. By using calcium indicators, optical mapping can also be used to directly image calcium transients, the rise and fall of intracellular calcium concentration that initiates the contraction and relaxation of cardiac muscle cells^10,11^. These features make optical mapping an extremely powerful pre-clinical technique for studying arrhythmogenesis and the electrophysiological effects of physiological, pathophysiological, and pharmacological stimuli.

Here, we provide a large database of cardiac optical mapping data from intact mouse and guinea pig whole hearts, and isolated mouse left atria. In total, 45 optical mapping datasets and over 400 individual files are provided. The dataset contains high resolution data obtained at multiple physiologically relevant pacing frequencies, both at baseline and in response to pharmacological agents (flecainide, carbenoxolone, ibutilide, cyclopiazonic acid, HMR 1556, and noradrenaline) or physiological interventions (ischaemia-reperfusion and sympathetic nervous stimulation). The data provided map cardiac electrophysiology both in sinus (intrinsic) rhythm and epicardial pacing, and during arrhythmic phenomena such as alternans and ventricular fibrillation.

Due to their high spatio-temporal nature, the datasets produced by optical mapping are large and complex. The data can also be limited by poor signal quality or corruption by motion artefacts^5,12^. Therefore, the database described here, as well as providing an ideal tool for high-resolution regional integration of the interventions applied, will act as a resource to understanding of optical mapping data, detailed mapping analysis and potential limitations of the technique. Our group has previously designed, validated and released an open-source software ElectroMap for analysis of optical mapping^13^. Several other open-source tools are also available^14–17^, including specialised software to analyse fibrillation dynamics^18^ and alternans behaviour^19^. These tools allow effective exploration of the optical mapping data provided, without significant prior expertise. Furthermore, the database we provide represents a freely available resource for development, testing and validation of novel mapping algorithms to further improve analysis capabilities.

## Methods

### Animal welfare ethics declarations

All experiments were undertaken in accordance with ethical guidelines set out by the United Kingdom Animals (Scientific Procedures) Act 1986 and Directive 2010/63/EU of the European Parliament on the protection of animals used for scientific purposes. Studies conformed to the Guide for the Care and Use of Laboratory Animals published by the U.S. National Institutes of Health under assurance number A5634-01. Studies were approved by the UK Home Office and relevant ethical committees at King’s College London (guinea pig, PPL: PF75E5F7F) and University of Birmingham (mouse, PPL: PPL 30/2967 and PFDAAF77F) respectively.

### Guinea pig whole heart optical mapping

Optical mapping of the innervated guinea pig whole heart was conducted as previously described (n = 23), where the entire rib cage is resected rather than just the heart^11,20,21^. This approach leaves intact the cardiac autonomic innervation, enabling stimulation of the sympathetic nervous system via the spinal cord as described below.

**Fig. 1:**
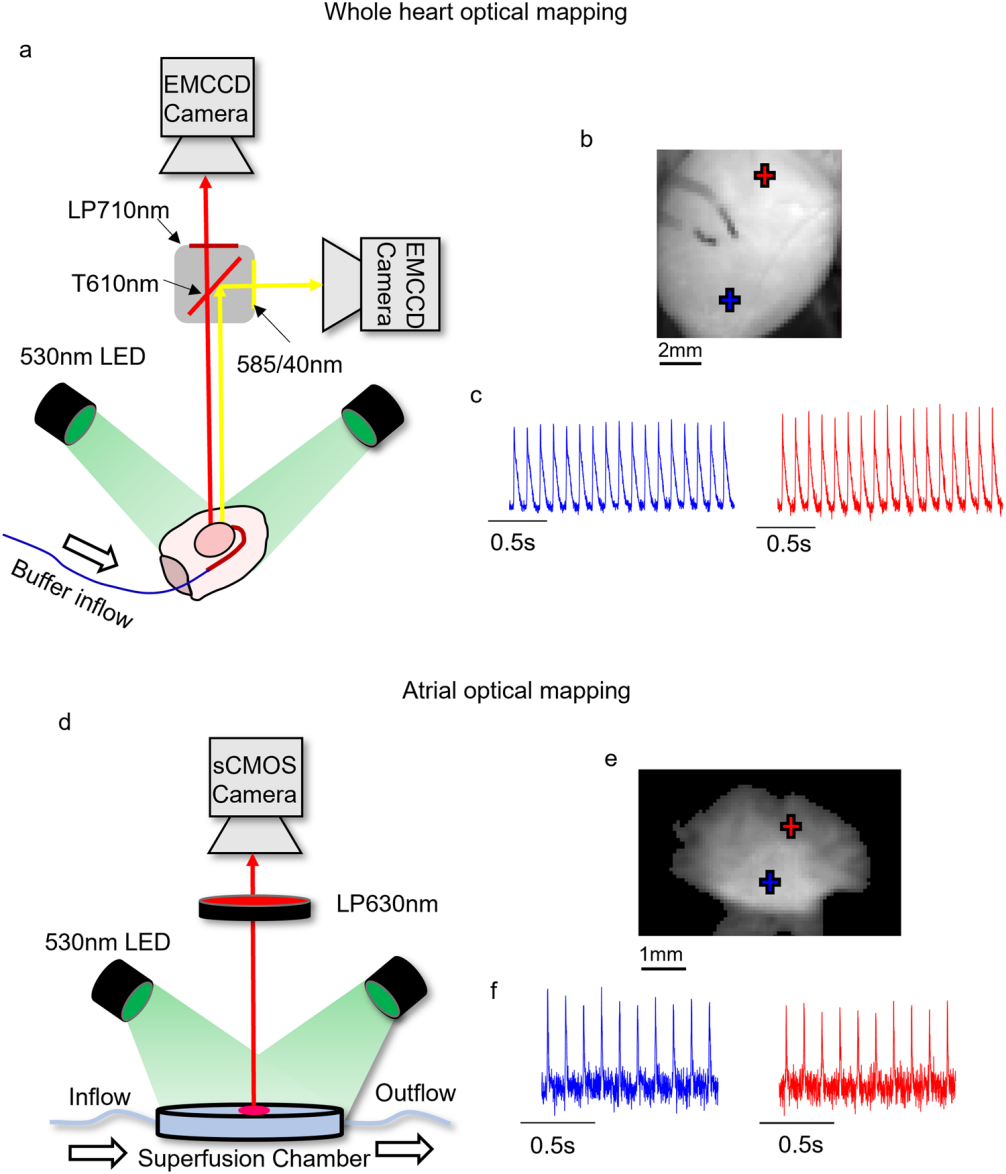
Optical mapping experimental setups. (**a**) Schematic diagram of optical mapping setup used for guinea pig and mouse whole heart optical mapping data in this dataset. (**b**) Fluorescent image of mouse whole heart loaded with di-4-anepps. (**c**) Example signals from marked locations collected from di-4-anepps loaded heart. (**d**) Schematic diagram of optical mapping setup used for mouse atrial optical mapping data in this dataset. (**e**) Fluorescent image of mouse atria loaded with di-4-anepps. (**f**) Example signals from marked locations collected from di-4-anepps loaded atria. LP = Long pass, T = Transmission, EMCCD = Electron multiplied charged coupled device, sCMOS = Scientific complementary metal oxide semiconductor.

Adult male guinea pigs were anesthetised using sodium pentobarbitone (160 mg/kg, intraperitoneal injection). Following confirmation of deep anaesthesia, an incision was made below the diaphragm. The rib cage was then removed, allowing isolation and cannulation of the descending aorta, and ligation of subclavian and carotid vessels. The preparation was then transferred to the optical mapping setup (Fig. 1a) where the heart was Langendorff perfused via the descending aorta. Hearts were perfused with warmed and oxygenation (37 °C, 95% O_2_/5% CO_2_) buffer solution at a pressure of 65–75 mmHg. Buffer solution contained in mM: NaCl 114, KCl 4.0, CaCl_2_ 1.6, NaHCO_3_ 24.0, MgSO_4_ 1.0, NaH_2_PO_4_ 1.1, glucose 11, sodium pyruvate 1 and decamethonium bromide 0.01. Hearts were mechanically uncoupled with blebbistatin (15 µM) to prevent motion artefacts. Where appropriate, pharmacological agents (see data records), were added directly into the perfusate following baseline recordings. Sympathetic nervous stimulation (SNS) was achieved by bi-lateral stimulation of efferent sympathetic nerves by a decapolar 5- French catheter in the spinal column. The catheter was inserted up to the 5–7th cervical vertebrae. The distal poles of the catheter were excited by pulse stimulation (40 V, duration 2 ms). Electrical stimulation frequency was varied between 3–8 Hz following testing at the beginning of each experiment to achieve a steady state heart rate of 320–340 bpm during SNS.

Langendorff perfused hearts were loaded with potentiometric (transmembrane voltage) dye di-8-anepps (1 mg/ml in DMSO; 200–300 μL) or rh-237 (2.5 mg/ml in DMSO; 25–50 μL) for action potential recording, Fig. 1a–c. For dual recording of action potentials and calcium transience, rh-237 was combined with intracellular calcium indicator rhod-2AM (200 μg dissolved in 200 μL DMSO + 20 μL 20% pluronic F-127). Hearts were excited by LED illumination at 530/50 nm, and emission light collected at >610 nm for di-8-anepps, >710 nm for rh-237 and 585/40 nm for rhod-2. Emission photons were imaged by Evolve Delta 512 × 512 pixel EMCCD cameras (500 Hz sampling rate, 64 × 64 pixels, 320 µm/pixel). During recordings, hearts were paced from left ventricular epicardial apex through silver bipolar pacing wires. Hearts were paced with a ramp protocol, starting at 160 ms pacing cycle length (PCL). PCL was then reduced by 10 ms every 10 beats until induction of ventricular fibrillation (VF).

### Murine whole heart optical mapping

Optical mapping of the mouse whole heart was conducted using a modified version of the above protocol as previously described (n = 12)^22^. Adult male mice were terminally anesthetized with isoflurane (4–5% in 100% O_2_, 1.5 L/min). Hearts were Langendorff perfused with warmed and oxygenated carbonated (37 °C, 95% O_2_/5% CO_2_) buffer solution at a pressure of 70–80 mmHg. Buffer solution contained in mM: NaCl 114, KCl 4, CaCl_2_ 1.4, NaHCO_3_ 24, NaH_2_PO_4_ 1.1, glucose 11 and sodium pyruvate 1. Hearts were mechanically uncoupled with blebbistatin (15 µM) to prevent motion artefacts. Where appropriate, pharmacological agents (see data records), were added directly into the perfusate following baseline recordings. For ischaemia-reperfusion experiments, global low-flow ischaemia was induced by reduction of flow rate by 75% for 3 minutes, with flow rate returned to original levels for reperfusion.

Hearts were loaded by injection of rh-237 (1.25 mg/mL in DMSO) into the perfusion line over a 5-minute period. Hearts were illuminated at 530/50 nm and emitted light collected at >710 nm. Emission photons were imaged by Evolve Delta 512 × 512 pixel EMCCD cameras (1000 Hz sampling rate, 51 × 51 pixels, 156 µm/pixel). During recordings, hearts were paced from the left ventricular epicardial surface through silver bipolar pacing wires at a PCL of 110 ms. Recording time was 5 seconds.

### Murine left atrial optical mapping

Murine left atrial optical mapping experiments were conducted as previously reported (n = 10, 6 Male, 4 Female)^13,23,24^. Adult mouse hearts were excised under deep terminal isoflurane-induced anesthesia (4–5% in O_2_, 1.5 L min^−1^). Whole hearts were isolated and Langendorff perfused via the ascending aorta at a flow rate of 2–4 mL/minute with standard bicarbonate buffered Krebs–Henseleit solution containing in mM: NaCl 118; NaHCO_3_ 24.88; KH_2_PO_4_ 1.18; Glucose 11.0; MgSO_4_ 0.83; CaCl_2_ 1.80; KCl 3.52 (37 °C, 95% O_2_/5% CO_2_). Potentiometric dye, di-4-anepps (0.125 mg/mL), was loaded over 5–10 minutes via bolus injection into the perfusion line. The left atrium was then removed, pinned in the superfusion chamber, and transferred to the optical mapping setup (Fig. 1d). In the optical mapping setup, atria were superfused with oxygenated Krebs–Henseleit solution (37 °C, 95% O_2_/5% CO_2_), and blebbistatin (42.75 µM) was added to the superfusate for mechanical uncoupling. Where appropriate, pharmacological agents (see data records), were added directly into the superfusate following baseline recordings. Atria were paced using platinum bipolar electrodes, initially at 1000 ms or 330 ms PCL for 10 minutes in dark conditions to allow equilibration.

For recordings, four LEDs at 530/50 nm illuminated the atria and filtered fluorescence (>630 nm) was collected using ORCA flash 4.0 sCMOS camera. Images were collected at 1000 Hz sampling rate with a maximal resolution of 200 × 2048 pixels (71 µm/pixel). Atria were paced with one of two ‘ramp’ protocols. Atria were paced at either 150 ms or 120 ms, and PCL was reduced by 10 ms every 20 stimuli down respectively. Recording time was between 30 seconds and 1 minute.

### Data analysis

All data analysis performed herein used the ElectroMap software. ElectroMap configuration files are provided with all datasets to recreate analysis outlined, and detailed analysis instructions are available within our previous publications^13,25^. Raw data were pre-processed by application of a 3 × 3 Gaussian spatial filter (standard deviation σ = 1.5). When required, baseline correction was performed using a top-hat kernel (200 ms length for guinea pig data, 100 ms for mouse)^26^. Automated PCL identification was applied, and the last 10 beats at respective PCLs were ensemble averaged to improve signal quality (other than for alternans and optical wave similarity (OWS) analysis, where single beat analysis is required).

Action potential duration to 80% (APD80) was calculated as the time from maximum upstroke velocity to 80% repolarisation. Activation time was defined as the midpoint of depolarisation. Conduction velocity (CV) was derived from activation maps using an implementation of the multi-vector method of Bayly *et al*. with a 5 × 5 window size^27^. Heterogeneity in APD and CV was calculated as the difference between the 95^th^ and 5^th^ percentiles, divided by the median. APD80 alternans magnitude (ΔAPD80) was calculated as the absolute difference between APD80 of one action potential compared to the previous. OWS, as a measure of temporal regularity, was calculated as described previously in detail^21^. Briefly, OWS was calculated by measuring the cosine similarity between individual amplitude normalised optical action potentials at a given pacing cycle length. OWS was then defined as the weighted sum of the similarity between all optical action potentials in the sequence. The result is OWS approaches 1 for temporally stable signals, and 0 for temporally irregular signals^21^.

For dominant frequency mapping, the frequency spectrum of the signal was computed using fast Fourier transform, with a Hann window applied. Zero padding was applied to achieve a frequency resolution of 0.05 Hz, and the frequency range studied was from 0.5 to 50 Hz^28^.

### Statistical methods

All data are presented as mean ± standard error of the mean. Group differences or effects of interventions such as changes in pacing cycle length were studied using one-way or two-way ANOVA as appropriate, with Tukey or Sidak’s correction respectively. Significance was defined as an adjusted P value < 0.05. Specific statistical tests used for each analysis presented are given in the figure legend. All statistics were performed using Prism 9 (GraphPad Software, San Diego, California).

## Data Records

The data associated with this database are summarised in Table 1 and available from figshare^29^. All data are provided in.mat format, and organised so that each folder contains an individual experiment (i.e. whole heart or isolated atrium). All PCLs are within single experimental files. .txt are also provided that contain suggested settings for use in ElectroMap.

**Table 1:** Summary of data records available at figshare^29^.

Datatype	Number of experiments	Pixel Size (µM)	Sampling Rate (kHz)	Pacing cycle lengths (ms)	Recording duration (s)	Pixel count (x,y)	Intervention(s)
Mouse whole heart	12	156	1	110	5	51 × 51	Ischemia-Reperfusion (n = 12)
							Flecainide, 1,2,3 µM (n = 6)
							Carbenoxolone, 10,30,50 µM (n = 6)
Mouse Atria	10	71.4	0.987	120-80	30–60	45 × 45*	Time control, 35 minutes (n = 5)
							Flecainide 1,5 µM (n = 5)
Guinea Pig Whole heart	23	320	0.5	170-80	60	64 × 64	Sympathetic Nervous Stimulation (n = 23)
							Ibutilide 100 nM (n = 5)
							Cyclopiazonic acid 10 µM (n = 4)
							HMR 1556 2 µM (n = 4)
							Noradrenaline 200 nM (n = 4)

*For mouse atrial files, pixel count varies between experiments.

## Technical Validation

To validate the optical mapping data presented in this dataset, we performed baseline recordings, pharmacological and physiological interventions with known effects on cardiac electrophysiology and time control experiments to ensure signal stability over time in absence of intervention.

### Guinea pig whole heart validation: cardiac restitution

To validate the data collected in the innervated guinea pig heart, we first quantified APD and CV as a function of PCL. As heart rate increases, (e.g. PCL decreases), the action potential duration in larger mammals such as the guinea pig shortens by modulation of several ionic mechanisms to enable a sufficient diastolic interval^30^. A further response to a decreased PCL is a reduced conduction velocity, primarily caused by a reduced availability of sodium channels which remain inactive following the previous excitation^31,32^. Collectively, the response of the cardiac tissue to heart rate/PCL is termed cardiac restitution.

**Fig. 2:**
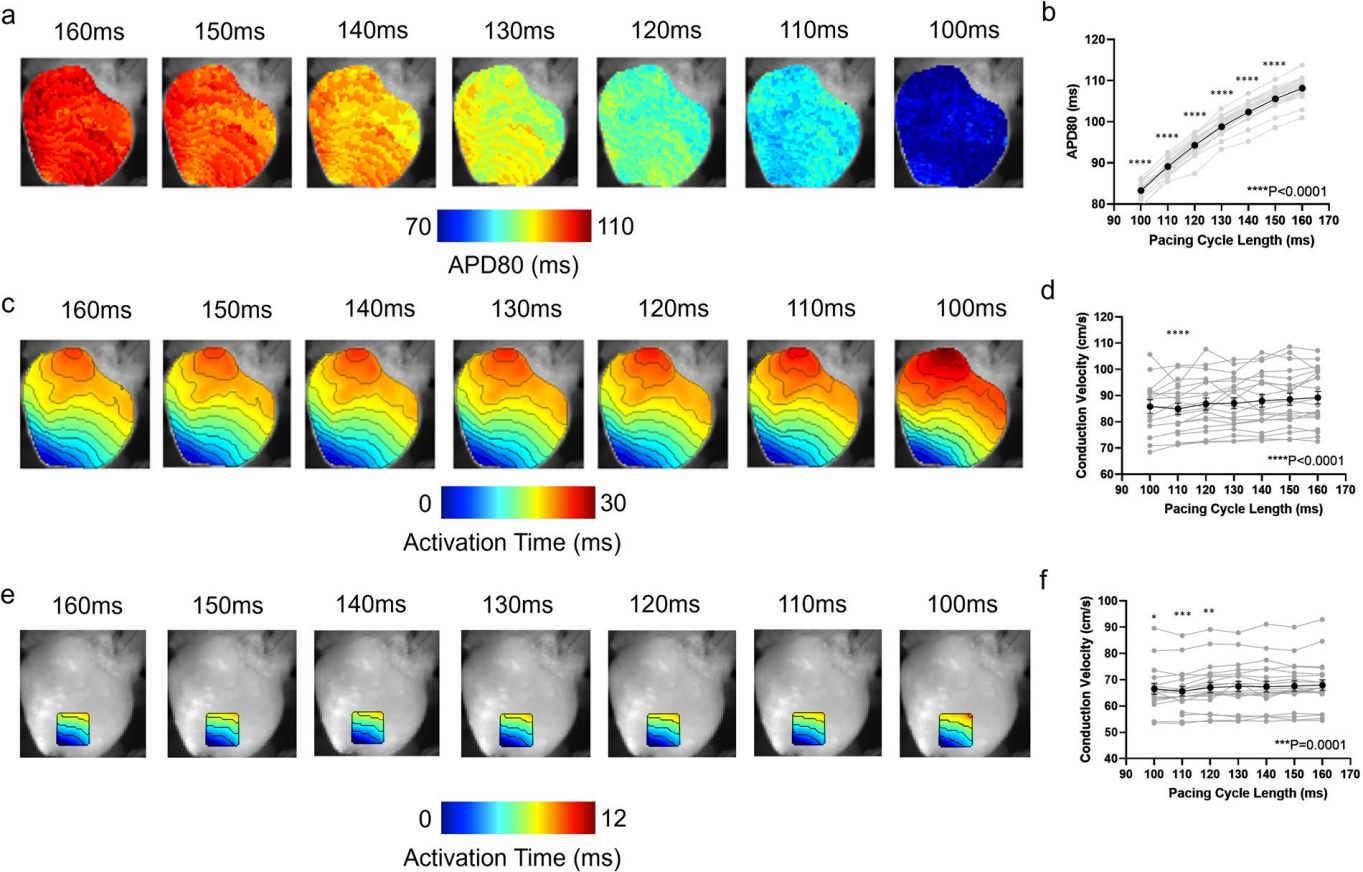
Restitution dynamics of guinea pig heart. (**a**) Example action potential duration 80 (APD80) images from guinea pig whole heart at decreasing pacing cycle lengths (PCL). (**b**) Grouped data of APD80 as a function of PCL. (**c**) Example activation images from guinea pig whole heart at decreasing PCLs. (**d**) Grouped data of conduction velocity as a function of decreasing PCLs. (**e**) Example activation images from guinea pig whole heart at decreasing PCLs with analysis area restricted to an area at the apex of the heart. (**f**) Grouped data of conduction velocity as a function of decreasing PCLs with restricted analysis area. n = 20. One-way ANOVA. ***P < 0.001, ****P < 0.0001 for overall interaction of APD80/CV with PCL, shown above x-axis. Multiple comparisons are for each PCL against the slowest PCL (160 ms). Significance (*P < 0.05, **P < 0.01, ***P < 0.001, ****P < 0.0001) are shown above respective PCL where a significant difference versus 160 ms pacing was identified.

Figure 2 demonstrates these expected restitution dynamics in the guinea pig data presented here. As PCL is decreased during the ramp protocol, APD80 decreases, Fig. 2a,b. For example, at a PCL of 160 ms, mean APD80 is 108.2 ± 0.63 ms, which decreases to 89.1 ± 0.57 ms when PCL is 110 ms (P < 0.0001). Equally, CV decreases as a function of decreased PCL, both when measured from the whole heart surface (Fig. 2c,d) and from a defined area near the apical pacing site (Fig. 2e,f). At a PCL of 160 ms, mean CV across the ventricular is 89.3 ± 2.4 cm/s, which decreases to 84.9 ± 2.1 cm/s when PCL is 110 ms (P < 0.0001). Similarly, when analysis is restricted to an area near the pacing site, CV slows from 68.0 ± 2.1 cm/s at 160 ms PCL to 65.6 ± 1.8 cm/s at 110 ms PCL (P = 0.0002). The changes in measured CV values depending on analysis area are discussed below (see *Usage notes*). Collectively, these data validate the pacing frequency response of the guinea pig data in this dataset.

### Guinea pig whole heart validation: ibutilide-induced action potential prolongation and sympathetic nervous stimulation -induced shortening

**Fig. 3:**
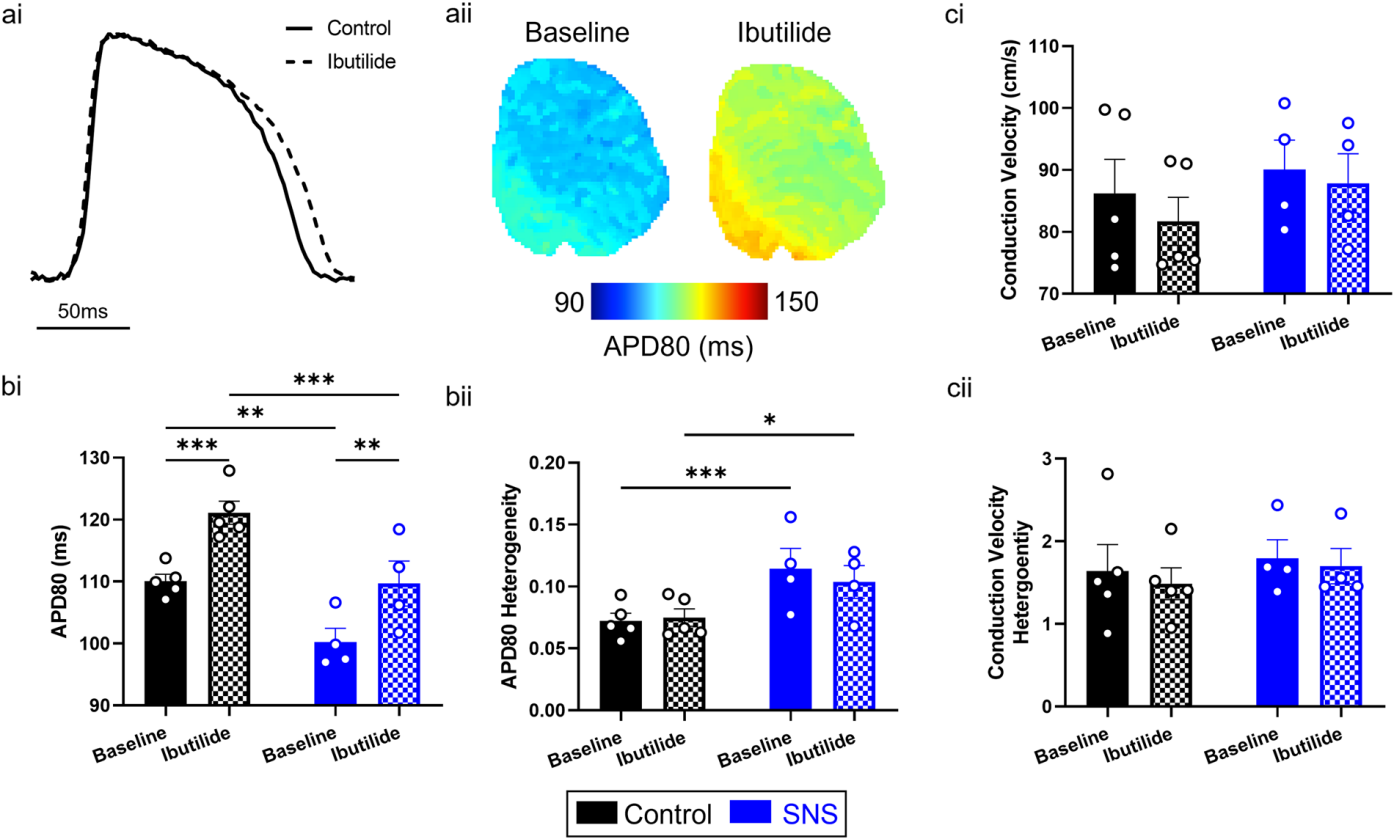
Effects of ibutilide and sympathetic nervous stimulation (SNS) in guinea pig heart. (**a**) Example optical action potential recordings **(i)** and action potential duration 80 (APD80) maps **(ii)** in control conditions and following ibutilide treatment. (**b**) Grouped data of APD80 (i) and APD80 heterogeneity (ii) before and after ibutilide treatment, both without (black) and with (blue) SNS. (**c**) Grouped data of conduction velocity (i) and conduction velocity heterogeneity (ii) before and after ibutilide treatment, both without (black) and with (blue) SNS. n = 5 for control, 4 for SNS. Two-way ANOVA. *P < 0.05, **P < 0.01. Multiple comparisons are made between all Baseline and ibutilide treated groups, and between control and SNS. Pacing cycle length = 160 ms.

Ibutilide is a class III anti-arrhythmic drug that’s primary mechanism of action is blockade of the rapid component of the delayed rectifier potassium current, I_Kr_^33^. As such, it is expected to prolong action potential duration. To validate pharmacological modulation of action potential morphology in these data, we mapped APD80 in the guinea pig whole heart before and after administration of ibutilide (100 nM), Fig. 3a. As further validation, we also repeated these measurements with application of sympathetic nervous stimulation (SNS), which is known to shorten rather than prolong APD^11,34^.

Figure 3a,b demonstrates that, as expected, ibutilide prolonged APD in both control conditions and during application of SNS. In control conditions, ibutilide prolonged APD80 from 110.1 ± 1.1 ms to 121.1 ± 1.8 ms (P = 0.0008). Similarly, with application of SNS, ibutilide prolonged APD80 from 100.2 ± 2.2 ms to 109.7 ± 3.6 ms (P = 0.0055). Furthermore, SNS significantly shortened APD80 from 110.1 ± 1.1 ms to 100.2 ± 2.2 ms (P = 0.026). Ibutilide-induced effects were homogeneous across the ventricular surface (Fig. 3bii), however SNS did significantly increase APD heterogeneity from 0.0722 ± 0.006 to 0.145 ± 0.016 (P = 0.0007). Again, these results are expected owing to the heterogenous distribution of cardiac neurons^34^. Figure 3c reveals neither ibutilide nor SNS significantly altered conduction dynamics. These results validate pharmacological and physiological induced perturbations of action potential morphology within these data.

### Guinea pig whole heart validation: pacing induced arrhythmia

**Fig. 4:**
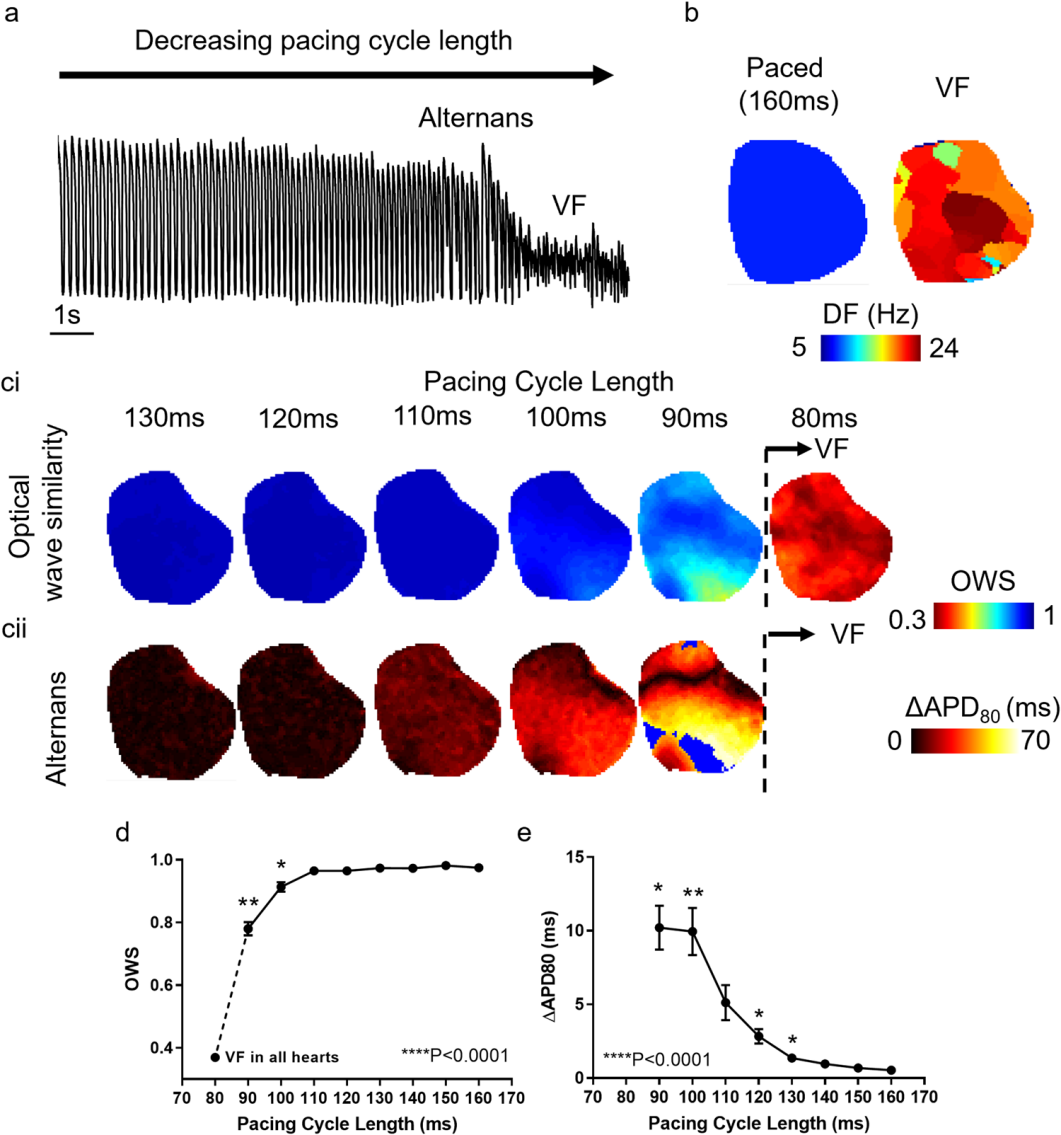
Ramp protocol induced arrhythmia in guinea pig whole hearts. **(a**) Example trace from guinea pig heart during ramp pacing protocol (initial pacing cycle length of 170 ms, which is decreased by 10 m every 20 stimuli until onset of fibrillation) inducing alternans and ventricular fibrillation (VF). (**b**) Example dominant frequency (DF) maps during physiological pacing and VF. (**c**) Example optical wave similarity (OWS, i) and action potential duration 80 alternans (ΔAPD80, ii) maps at decreasing PCLs. Note: blue areas in alternans maps denote areas where ΔAPD80 could not be calculated. (**d**) Grouped data showing OWS as a function of PCL and during VF. (**e**) Grouped data showing ΔAPD80 as a function of PCL. n = 6. One-way ANOVA. ****P < 0.0001 for overall interaction of OWS/ΔAPD80 with PCL, shown above x-axis. Multiple comparisons are for each PCL against the slowest PCL (160 ms). Significance (*P < 0.05, **P < 0.01) are shown above respective PCL were a significant difference versus 160 ms pacing was identified.

Rapid pacing of *ex-vivo* cardiac perpetrations is a common approach for induction of pro-arrhythmic phenomena such as cardiac alternans, and indeed arrhythmias such as atrial and ventricular fibrillation (AF/VF). To validate the use of these data for studying arrhythmogenesis, we applied dominant frequency, alternans (ΔAPD80) and optical wave similarity (OWS) analysis before and during onset of VF induced by the ‘ramp’ pacing protocol (Fig. 4a). Figure 4b demonstrates that during physiological pacing, the dominant frequency of the action potential signal is homogenous across the tissue and dictated by the pacing frequency. In contrast, during VF, the dominant frequency is greater and extremely heterogenous, as has been demonstrated in several previous studies^12,18^.

Figure 4c–e demonstrates the onset of temporal irregularity in the action potential signal at faster PCLs preceding (and during) VF onset. OWS (a measure of the morphological similarity of one action potential with all others at the same pacing frequency based on the whole waveform^21^) decreases at shorter PCLs, Fig. 4ci,d (P < 0.0001). For example, at a PCL of 160 ms, OWS = 0.98 ± 0.007 which decreases to 0.78 ± 0.021 when PCL decreases to 90 ms (P = 0.0066). During VF, OWS further reduces to 0.37 ± 0.02. Concurrently, APD alternans magnitude (a comparison of APD of successive action potentials) increases with decreased PCL, Fig. 4cii,e (P < 0.0001). At a PCL of 160 ms, ΔAPD80 = 0.53 ± 0.043 ms, which increases to 10.2 ± 1.50 ms at a PCL of 90 ms (P = 0.0108). ΔAPD80 could not be measured during VF due to the impracticality of defining APD during fibrillation. Taken together, these results validate the induction of temporal irregularity, alternans, and VF in the data provided in this dataset.

### Mouse whole heart validation: flecainide and carbenoxolone induced conduction slowing

Flecainide and carbenoxolone are two pharmacological agents known to slow cardiac CV by different mechanisms. Flecainide is an antiarrhythmic drug that primarily blocks sodium channels, which whilst prolonging the refractory period also leads to slowing conduction^35,36^. Carbenoxolone reduces gap junction coupling, and hence reduces the conductance between neighbouring cells, resulting in slowed action potential propagation^37^. To validate the provided data in the mouse whole heart, we therefore measured CV at increasing concentrations of flecainide and carbenoxolone.

**Fig. 5:**
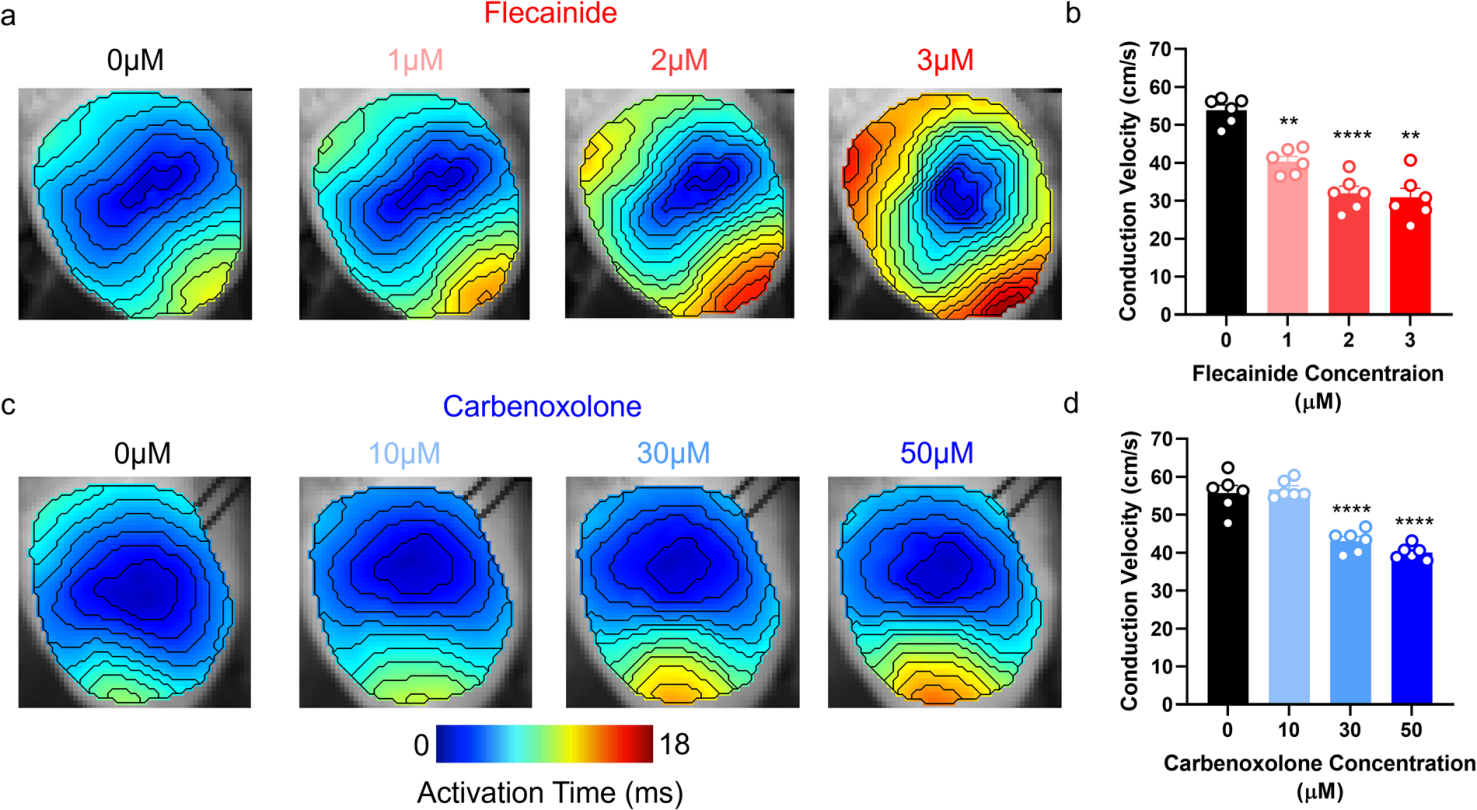
Flecainide and carbenoxolone induced conduction slowing in mouse whole hearts. (**a**) Example activation maps from mouse whole heart at increasing concentrations of flecainide. (**b**) Grouped conduction velocity data from mouse whole heart at increasing concentrations of flecainide. (**c**) Example activation maps from mouse whole heart at increasing concentrations of carbenoxolone. (**d**) Grouped conduction velocity data from mouse whole heart at increasing concentrations of carbenoxolone. n = 6 for both experiments. One-way ANOVA, **P < 0.01, ***P < 0.001, ****P < 0.0001 against 0 µM flecainide/carbenoxolone respectively. Pacing cycle length = 110 ms.

Figure 5 demonstrates, as expected, both agents slowed cardiac CV in a dose-dependent manner. In the case of flecainide, CV slowed from 53.86 ± 1.4 cm/s to 40.36 ± 1.3 cm/s (1 µM, P = 0.0016), 32.01 ± 1.8 cm/s (2 µM, P = 0.005) and 30.86 ± 2.4 cm/s (3 µM, P = 0012) respectively, Fig. 5a,b. 10 µM carbenoxolone did not significantly reduce CV. However, higher doses of carbenoxolone slowed CV from 55.75 ± 2.0 cm/s to 43.07 ± 1.1 cm/s (30 µM, P < 0.0001) and 40.07 ± 0.75 cm/s (50 µM, P < 0.0001) respectively, Fig. 5c,d. These results validate the pharmacological response of the mouse whole heart data presented herein.

### Mouse whole heart validation: acute ischaemia-reperfusion-induced conduction slowing and action potential shortening

Ischaemia-reperfusion injury exerts several effects on cardiac electrophysiology. Ischaemia (restriction of blood flow) results in metabolic stress, cell acidification and increased concentration of potassium in the extracellular space^38^. This can increase the resting membrane potential which reduces sodium channel availability, thereby leading to slowing of CV. Strong activation of K_ATP_ channels also causes severe APD shortening. Therefore, we measured CV and APD following acute (3 minutes) ischaemia in our mouse whole heart data, and following 3 minutes reperfusion, to validate the physiological response of the cardiac tissue in the data presented here.

**Fig. 6:**
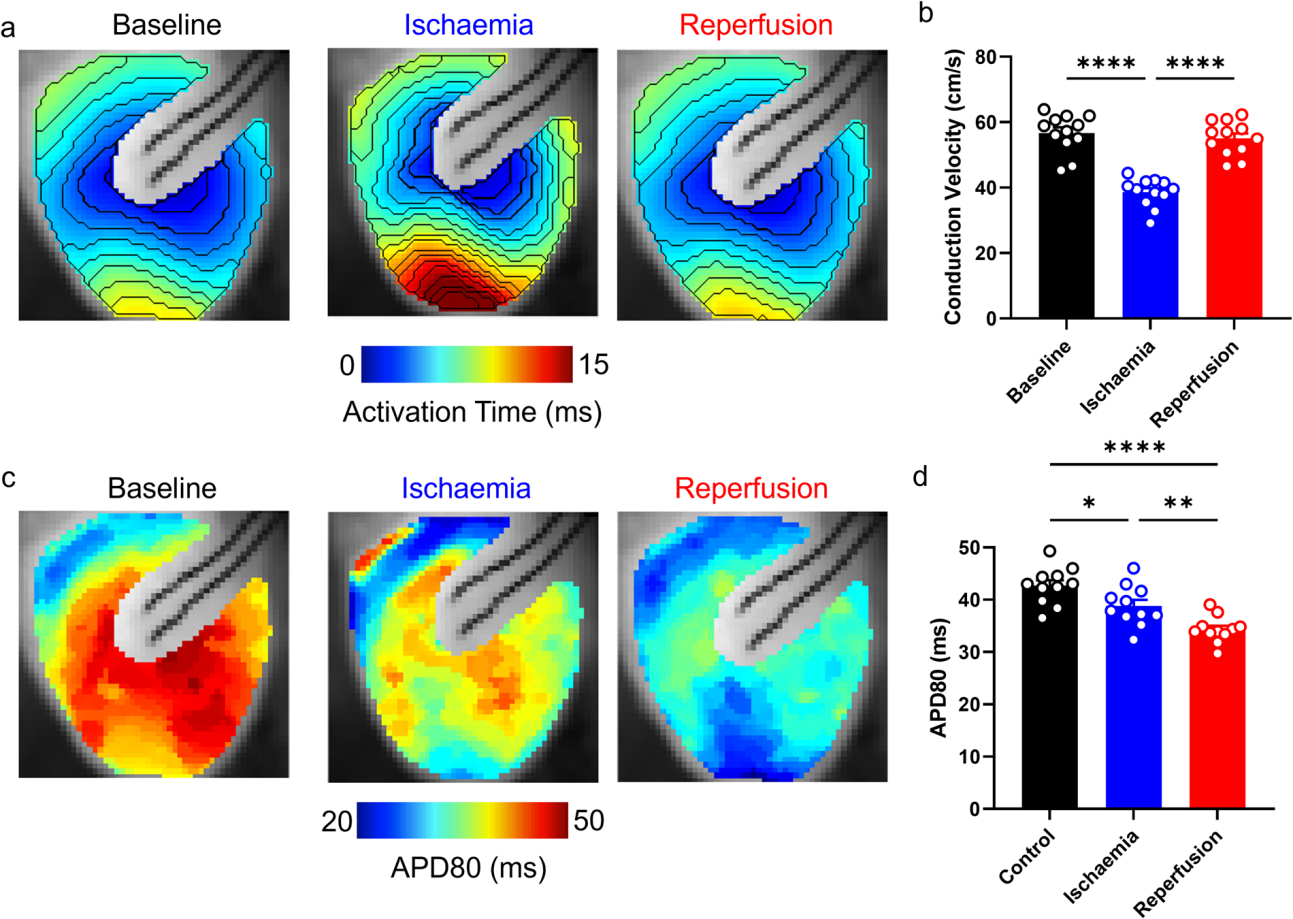
Ischaemia induced conduction slowing and action potential shortening in mouse whole hearts. (**a**) Example activation maps from mouse whole heart in before (black), during (blue, ischaemia) and after (red, reperfusion) low-flow ischaemia. (**b**) Grouped conduction velocity data from mouse whole heart in control, ischaemia and reperfusion conditions. (**c**) Example action potential duration 80 (APD80) maps from mouse whole heart at baseline (black), during (blue, ischaemia) and after (red, reperfusion) low-flow ischaemia. (**d**) Grouped APD80 data from mouse whole heart in control, ischaemia, and reperfusion conditions. n = 12. One-way ANOVA, *P < 0.05, **P < 0.01. ****P < 0.0001. Multiple comparisons are made between all groups (Baseline, Ischaemia, Reperfusion). Pacing cycle length = 110 ms.

Figure 6a,b demonstrates that, as expected, ischaemia reduced cardiac CV. CV reduced from 56.72 ± 1.7 cm/s in control conditions to 38.59 ± 1.24 cm/s following ischaemia (P < 0.0001). 3 minutes reperfusion restored CV to 55.04 ± 1.5 cm/s (P = 0.14 vs control, P < 0.0001 vs ischaemia). Furthermore, Fig. 6c,d demonstrates that ischaemia reduced APD80 from 42.68 ± 1.07 ms to 38.83 ± 1.15 ms (P = 0.0294). Unlike CV, reperfusion did not restore APD80 which further reduced to 34.28 ± 0.8 ms (P < 0.0001 vs control, P = 0.009 vs ischaemia). These results validate the physiological response to ischaemia-reperfusion of the mouse whole heart data presented here.

### Mouse isolated left atria validation: flecainide induced conduction slowing and time control data

**Fig. 7:**
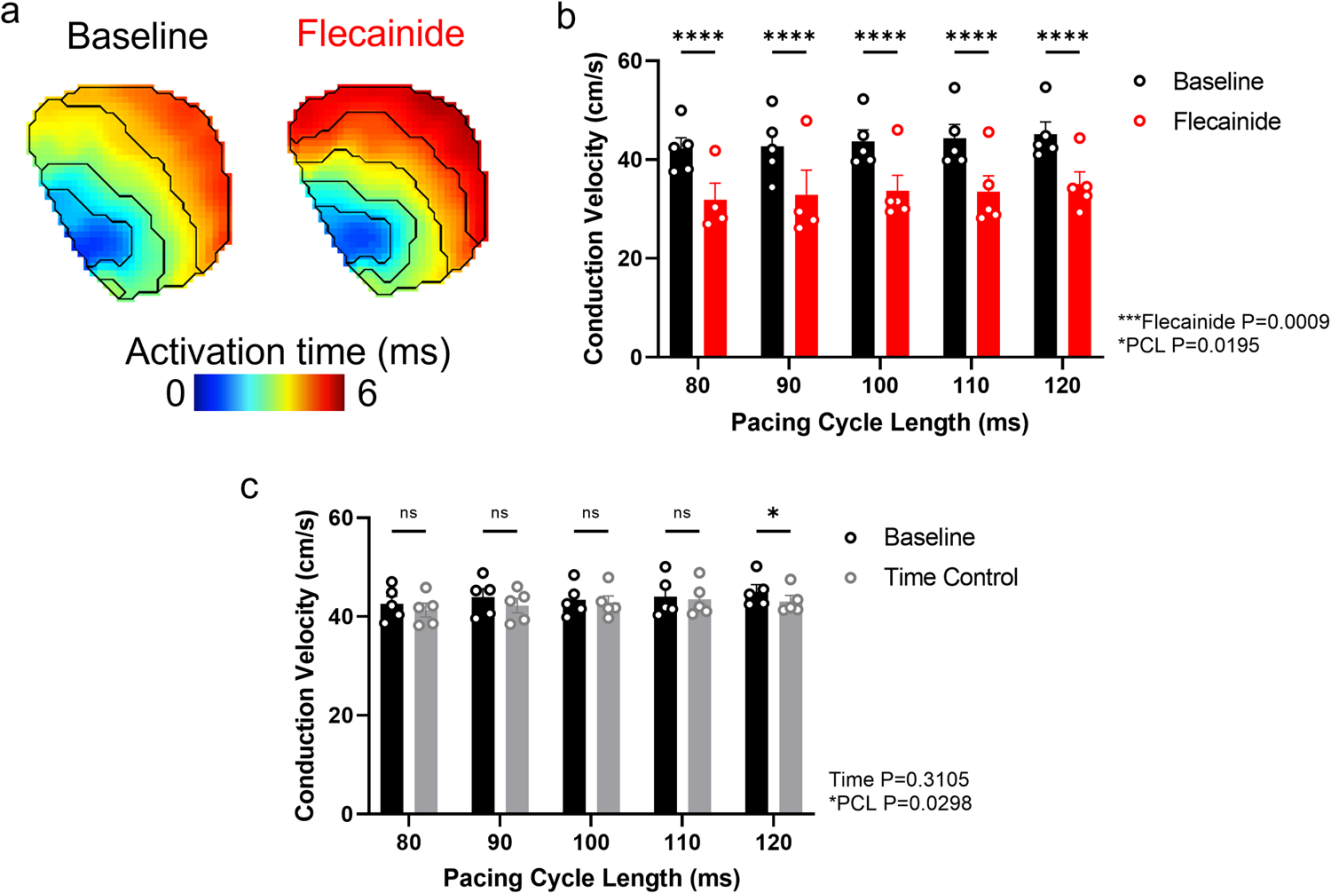
Flecainide-induced conduction slowing in mouse left atria. (**a**) Example activation maps at baseline and following 1 µM flecainide treatment of mouse left atria. (**b**) Group data of conduction velocity before and following 1 µM flecainide treatment of mouse left atria at a range of pacing cycle lengths (PCLs). **c)** Grouped data of conduction velocity at baseline and 20 minutes later from mouse left atria at a range of PCLs. n = 5. Two-way ANOVA, *P < 0.05, ***P < 0.001, ****P < 0.00001 between baseline and flecainide/time control respectively. Overall interaction of CV with PCL and flecainide/time is shown to the bottom right of the figures *P < 0.05, ***P < 0.001.

As stated above, flecainide is an antiarrhythmic drug that blocks sodium channels, slowing the onset of the action potential and thus slowing conduction^35^. Therefore, to validate our data generated in isolated mouse left atria, we measured CV at baseline and following 1 µM flecainide treatment. Figure 7a,b. As expected, flecainide significantly slowed CV at all PCLs tested (P = 0.0009). For example, at 100 ms PCL, flecainide slowed CV from 43.70 ± 2.3 cm/s to 33.73 ± 3.1 cm/s (P < 0.0001). Furthermore, as observed in the guinea pig data, decreased PCL slowed CV (P = 0.0195).

To validate the temporal stability of these preparations and drug-induced effects, we concurrently performed time control experiments. These were identical to the flecainide experiments in terms of experimental setup, recording time, pacing frequency, dye concentration and blebbistatin concentration, but did not include addition of 1 µM flecainide to the superfusate. Figure 7c demonstrates that over the experimental time (20 minutes) there is a small but non-significant reduction in CV (P = 0.3105). For example, at 100 ms PCL, 20 minutes on the experimental setup slowed CV from 43.39 ± 1.5 cm/s to 42.82 ± 1.4 cm/s (P = 0.927). These results validate the pharmacological response to flecainide of the mouse atrial data presented here.

## Usage Notes

As stated in the Methods, all data analysis provided here has been undertaken using ElectroMap software (https://github.com/CXO531/ElectroMap). For help with use of this data within ElectroMap, example setting configuration files are provided will all datasets than can easily be loaded into ElectroMap. For further instruction on use of this software, the reader is directed towards our previous publications^13,25^.

Furthermore, several other open software packages are also available which can be used with the provided data^14–19,39^.

### Limitations and considerations in optical mapping

There are several aspects of optical mapping experiments, and the data produced, that should be considered when using and interpreting these data and others. Firstly, effective recording of the action potential (or calcium transient) morphology requires uncoupling of the mechanical contraction of the heart (although progress is being made to realise optical mapping in the freely beating heart^12,40,41^). Commonly, this is achieved using excitation-contraction uncouplers, such as Blebbistatin in the data presented herein. The uncoupled heart lacks bidirectional interaction between mechanical and electrical activity (e.g. stretch activated ion channels^42^), and has reduced oxygen and energy demand^40^. There are also reports that Blebbistatin exerts direct effects on cardiac electrical activity^43^, although there are several reports to the contrary^12,44^. Nevertheless, the potential effects of mechanical uncoupling should be considered when interpreting optical mapping data. Further, there is evidence other compounds required for optical mapping, such as the voltage dye di-4-anepps, may exert effects on cardiac electrophysiology such as conduction slowing^45^.

Optically recording signals in intact tissue can alter signal morphology due to the interaction of tissue and light^22,46–48^. Although minimal, excitation light will propagate into the tissue, and excite fluorophores away from the imaged surface, typically the epicardium. Therefore, the recorded signals will be a sum of optically recorded action potentials/calcium transients from surface cells, but also to some extent from cells below in the myocardium (or opposite atrial wall in the isolated mouse atria preparations).

Another important aspect is the effect data analysis methods can have on data interpretation. For example, in these data we have applied the ‘depolarisation midpoint’ method to define activation time. The reason for this is that other methods, such as commonly used ‘maximum upstroke velocity’, are limited by sampling rate^49^. This reduces the tissue proportion in which effective CV analysis can take place, as large areas of the tissue will be measured to have simultaneous activation times (making effective CV infinity)^13^. This is particularly problematic in the murine atrial data presented herein, where the entire tissue activates in ca. 5 ms. However, previous work suggests the ‘time of maximum upstroke velocity’ of the optical action potential is the best measure of local activation time^50^, despite the issue of sampling rate. Furthermore, several studies demonstrate the recorded optical amplitude and upstroke morphology, from which the ‘depolarisation midpoint’ and ‘time of maximum upstroke velocity’ are derived, is altered by asynchronous activation times, transmural propagation direction, optical scattering and pixel integration from several cells^22,47,48^. Within the ElectroMap software, we therefore include both these methods for activation time calculation (and more) for use with these data and others.

Finally, it is also important to consider underlying conduction dynamics of intact tissue when quantifying parameters such as CV in optical mapping datasets. For example, when conduction analysis is applied across the entire imaged surface, the baseline (defined as slowest PCL measured without intervention) ventricular CV of the mouse heart (e.g., 55.04 ± 1.5 cm/s, Fig. 6) is measured to be markedly slower than that of the guinea pig (89.3 ± 2.4 cm/s, Fig. 2c,d). However, a potential explanation for the increased CV observed in the guinea pig heart here is the increased size of the guinea tissue and pacing from the apex of the heart. The further from the pacing site, the more likely that propagation is no longer parallel to the epicardial surface due to fibre orientation in the heart and potential activation of conduction pathways such as the Purkinje network. This phenomenon, often termed ‘epicardial breakthrough’, can distort activation patterns as observed at the epicardial surface, artificially increasing measured CV values^22,46^. Indeed, in Fig. 2c the isochronal lines spread as the activation wave moves away from the pacing site suggesting epicardial breakthrough. In contrast, in the smaller mouse tissue paced from the centre, this phenomenon is not observed (Fig. 5a,c). In support of the vastly increased CV of the guinea pig heart compared to the mouse being an artefact of transmural conduction patterns, when analysis area is restricted to near the apical pacing site (where conduction is assumed to be more parallel to the epicardial surface), guinea pig CV is more closely matched to the mouse at 68.0 ± 2.1 cm/s, (Fig. 2e,f), although remains quicker.

## Data Availability

All code used for analysis of the data are freely available at: https://github.com/CXO531/ElectroMap.
