# Publisher Correction: High resolution optical mapping of cardiac electrophysiology in pre-clinical models

**DOI:** 10.1038/s41597-024-02941-w

**Published:** 2024-01-18

**Authors:** Christopher O’Shea, James Winter, S. Nashitha Kabir, Molly O’Reilly, Simon P. Wells, Olivia Baines, Laura C. Sommerfeld, Joao Correia, Ming Lei, Paulus Kirchhof, Andrew P. Holmes, Larissa Fabritz, Kashif Rajpoot, Davor Pavlovic

**Affiliations:** 1 Institute of Cardiovascular Sciences, University of Birmingham, Birmingham, UK; 2 Heart Center, Department of Clinical and Experimental Cardiology, Amsterdam UMC, location AMC, Amsterdam, The Netherlands; 3 William Harvey Research Institute, Queen Mary University of London, London, UK; 4 Department of Pharmacology, University of Oxford, Oxford, UK; 5 Department of Cardiology, University Heart and Vascular Centre, University Medical Center Hamburg-Eppendorf, Germany and German Center for Cardiovascular Research (DZHK) partner site Hamburg/Kiel/Lubeck, Lubeck, Germany; 6 Institute of Clinical Sciences, University of Birmingham, Birmingham, UK; 7 University Center of Cardiovascular Science, UKE, Hamburg, Germany; 8 School of Computer Science, University of Birmingham, Birmingham, UK

**Keywords:** Arrhythmias, Heart failure, Arrhythmias, Heart failure

Correction to: *Scientific Data* 10.1038/s41597-022-01253-1, published online 31 March 2022

The DOI URL for the main data citation (reference 29, for figshare) was incorrect in the original version. This has been updated in the pdf and HTML versions of the paper to the correct link (10.6084/m9.figshare.c.5700466.v1).

